# Everybody's Page

**Published:** 1889-05-18

**Authors:** 


### EVERYBODY'S PAGE.

LEPROSY is increasing in Russia. During the last ten years 49 patients were treated in the St. Petersburg hospitals, half of whom were natives of the city. The Baltic provinces suffer most from the disease.

BARON F. VON MUELLER, the president of the section of pharmacology at the Inter-Colonial Medical Congress held at Sydney, recommended the cultivation of cinchona in East Australia, the frostless, temperate mountain regions of which are admirably fitted for its successful cultivation.

THE Municipality of Berlin intends to create a new establishment for epileptics at Bisdorf, a village near the city. It is intended to hold 700 patients, but may be enlarged to receive 1,000, and is to have a farm or ample grounds attached to it. It will consist of a central building and a number of cottages, each with a garden round it.

HERE is the advice given in a paper called *The Writer* to literary aspirants. It is headed, "How to Send Manuscripts": "The best way to send manuscripts is in a stout envelope long enough to hold commercial note sheets without folding. A similar envelope, stamped and addressed for return of manuscript, should be enclosed. Before setting out to post a manuscript, always commit suicide." Yet some of us have done it—and survived.

THE qualities that make pilocarpine a useful remedy in diptheria are common to the garden tulip and the lily of the valley, and the claims of the latter are supported by no less an authority than Dujardin-Beaumetz. It is not, however, a new claimant to the honours of the pharmacopœia. More than 300 years ago Matthioli, the physician of the Emperor Maximilian, insisted on its value as a cardiac sedative, while See and other physicians placed it next to digitalis in mitral diseases.

IT is a mistake to look upon milk as a beverage. It is a liquid food, and though it quenches thirst at the moment, it makes it more intense after it has been some time in the stomach, and its digestion has commenced. Healthy infants who receive a sufficiency of milk often cry for long periods, to the bewilderment and distress of mothers and nurses, simply because they are thirsty; and in many cases where indigestion is caused by weakness of insufficiency of the gastric juice, the child would be greatly benefited by a drink of water.

IN a Western American city lived Mrs. R——, a lady teacher and practitioner of "Christian science," or faith-healing, which effects its cures by the faith of the operator, not of the patient. One day Mrs. R—— noticed passing her window a man who limped severely, as though suffering from acute rheumatism. Pity, and perhaps the desire to perform a cure which a sceptical world could not say was due to imagination, moved her to apply her healing powers without his knowledge. In a day or two she had the satisfaction of seeing the limp grow less perceptible, and before long it almost entirely disappeared. She told the story to her class, but was surprised and grieved to see a quiet smile of incredulity pass round her pupils at the narration. After the lesson was over, one who knew her intimately remained behind to say: "Oh, Mrs. R——, don't tell that story again. The patient was Mr. L——, whom we all know pretty well. He has a cork leg. A little while ago it got out of order, and it was several days before he could get it mended, and several more before a new one came from Sharp and Smith's in Chicago." But Mrs. R—— still believes in faith-healing.

AMONG the Romans a man could divorce his wife if she was childless.

NATIVE sulphate of baryta is a sure poison for rats and mice, and one which, when mixed with lard, they seem to like greatly.

DR. H. MACNAUGHTEN JONES has invented antiseptic cigarettes for fumigating the naso-pharynx, in order to destroy various disagreeable growths. The main constituents are eucalyptus and idioform.

THE function of a negro's black skin is supposed to be the conversion of the sun's light into heat. The heat thus generated remains in the skin, and does not penetrate to the deeper tissue. Being thus provided with a sun-proof armour, the negro can stand an amount of heat that would be fatal to a white man, and he runs hardly any risk of sunstroke.

THE merit claimed for sulphonal is that it is a hypnotic which is free from danger, and singularly effectual when the insomnia arises from purely nervous irritability. The sleep it produces is more normal in character, and more prolonged than that which results from the use of chloral, and it has no after effects that injure the organs either of digestion or respiration.

THE editor of the *New York Medical Times* says that the worst case of septicaemia he ever saw was cured by alcohol. For three weeks the patient was nourished on whiskey and water exclusively, and was constantly bathed in alcohol, with which also the dressings used in the case were moistened. Other antiseptic measures were employed, but the alcohol was the main feature of the treatment, and the patient made a good recovery.

MANY scientific men believe that aluminium is destined to replace iron as the metal of the future. It is plentiful; indeed, it is to be found in every bank or bed of clay, but till lately, when the electric current was used for the purpose, the cost of reducing it to the metallic form was so large as to be prohibitive. Aluminium is a soft white metal; it never rusts, it weighs little more than a third as much as iron; it combines easily with other metals, and when alloyed with copper it is harder than steel.

IN Tracadie, in the Province of New Brunswick, there is a lazaretto for lepers. It contains sixteen lepers, nine male and seven female. who are nursed by sisters of charity. The disease exists in only two counties in the province, and is seen particularly along the shores of the Baie des Chaleurs, the Gulf of St. Lawrence, and the Miramichi river. The first case ever heard of was that of Ursule Landry, a woman who died in 1828, after suffering from ulcerating leprosy for nine years. It was supposed that in her case the disease was hereditary, for though she and her father were born in the province, her grandfather came from Normandy at a time when leprosy was prevalent there. The disease having spread, the Government established a hospital in 1844, and tried to force the lepers to occupy it. But this was not easily done. Families concealed their leprous relatives, and taught them to hate the idea of a public institution. It was only after the Government gave up compulsion, and a band of sisters from Montreal volunteered their services, and made the hospital a home, that the lepers consented to occupy it. But now almost every leper in the district is there; and as, once admitted, they remain in the asylum for life, there is every chance of the disease being stamped out.

